# Combining organic fertilizer with soil conditioner alleviates continuous cropping obstacles in protected cowpea by improving soil properties and microbiome

**DOI:** 10.1038/s41598-025-27630-5

**Published:** 2025-11-26

**Authors:** Dexin Wang, Yang Chen, Xiaoying Yang, Chunfeng Wan, Zhenzhen Zhang, Jianing Xu, Tao Xue, Chen Bo

**Affiliations:** 1 College of Agriculture and Bioengineering, Heze University, Heze, 274015 China; 2 Anhui Provincial Engineering Laboratory for Efficient Utilization of Featured Resource Plants, College of Life Sciences, Huaibei Normal University, Huaibei, 235000 China; 3 Editorial Department of Journal of Heze University, Heze University, Heze, 274015 China; 4 School of Biology and Medicine, Shandong University of Technology, Zibo, 255049 China

**Keywords:** Organic fertilizer, Soil conditioner, Protected cowpea, Soil nutrients, Microbial community, Ecology, Ecology, Environmental sciences, Microbiology, Plant sciences

## Abstract

Cowpea (*Vigna unguiculata*) is a nutritious vegetable and a key crop in China, encompassing a sizeable cultivation area. However, continuous cropping has led to declines in yield and quality, limiting sustainable development in the protected cowpea industry. This study investigated the effects of combining organic fertilizer with soil conditioner (an amendment containing beneficial microbes and organic components) on protected cowpea production, soil physicochemical properties, and microbial community structure in soils subjected to five consecutive cowpea cropping cycles. The results demonstrated that combined application significantly improved cowpea yield and quality. Specifically, soil pH decreased by 5.4%, while soil organic matter (OM) content increased markedly, especially during the vegetative growth stage, displaying a 70.4% increase. The activities of key soil enzymes, including sucrase (SC), protease (PT), nitrate reductase (NR), dehydrogenase (DHO), and phosphatase, were enhanced, while the urease (UE) activity decreased. Additionally, the combined application improved bacterial and fungal abundance and diversity. Operational taxonomic unit (OTU) richness exhibited positive correlations with various soil nutrient indicators, enzyme activities, and physicochemical properties. Moreover, cowpea protein, carbohydrate, and energy contents were positively correlated with specific soil enzyme activities and nutrient levels. The combined treatment increased the cowpea yield by nearly 32,849 kg per ha and protein content by 32.3% compared with applying only organic fertilizer while optimizing the soil microbial community, improving soil structure and fertility, and effectively mitigating the issues of continuous cropping in protected cowpea production.

**Supplementary Information:**

The online version contains supplementary material available at 10.1038/s41598-025-27630-5.

## Introduction

Cowpea (*Vigna unguiculata* (L.) Walp.) is a leguminous crop rich in protein, iron (Fe), phosphorus (P), calcium (Ca), vitamins, fiber, and other essential nutrients. In addition, it contains various essential amino acids, including tryptophan and lysine, contributing to its high nutritional value^1,2^. With the rapid expansion of protected agriculture, the cultivation area of protected cowpea has increased steadily. However, continuous cropping has weakened plant growth, resulting in significant declines in yield and quality and a sharp rise in pest and disease incidence, posing serious challenges for developing the protected cowpea industry^3,4^.

Continuous cropping of protected cowpea often leads to various soil-related issues such as compaction, degradation of soil aggregate structure, and surface crusts with white or green discoloration. These problems intensify within three to five successive planting cycles, causing an increase in soil-borne diseases and pests, restricted root development, stunted growth, reduced stress tolerance, and noticeable reductions in yield and quality^3,5^. In addition, repetitive cultivation weakens soil fertility and leads to the accumulation of allelopathic compounds released by roots, further inhibiting cowpea growth^6,7^. In severe cases, extensive seedling mortality may occur, resulting in large-scale yield losses or even total crop failure. Thus, continuous cropping is an urgent issue in protected cowpea production systems requiring resolution.

Recent research has identified soil organic matter (OM) as a key factor influencing soil physical and chemical properties, enzyme activity, crop yield, and quality^8,9^. Animal manure is a major source of OM and plays a vital role in enhancing soil carbon sequestration^10,11^. The long-term application of manure as a soil amendment profoundly affects the molecular composition of soil OM and the transformation processes of soil carbon. Moreover, manure application significantly reduces carbon losses from the soil by interacting with soil microbial communities^12^. Replacing chemical fertilizers with organic fertilizers improves soil quality by increasing nutrient levels, stimulating microbial biomass and abundance, accelerating soil biochemical processes, and promoting extracellular enzyme secretion^11^. The continuous application of organic fertilizers reduces the effects of climatic and edaphic variability on crop yield and strengthens the link between soil microbial function and crop productivity^13^. For example, the combined application of manure and nitrogen (N) fertilizer has been demonstrated to improve maize yield^14^, while manure application enhances P bioavailability through microbially mediated mechanisms, thus increasing cotton production^15^. These findings underscore the key role of manure in maintaining soil nutrient levels, improving soil structure, and regulating the microbial environment.

However, while organic fertilizers are crucial for nutrient supply and microbial stimulation, they may not fully address severe soil structural degradation or persistent pH imbalances common in continuous cropping systems. In this context, soil conditioners, often comprising beneficial microorganisms, organic components, and mineral amendments, offer a complementary approach. They can improve the overall efficacy of organic fertilizers and alleviate continuous cropping problems by improving soil aggregates, buffering pH, increasing nutrient utilization, introducing beneficial microbial activity, and creating a more favorable physical and chemical environment^16^. According to Yu et al. (2023), soil conditioners effectively increased the content of available potassium and organic matter in tobacco-cultivated soil, while also enhancing the abundance of beneficial microbial communities^17^. In continuous cucumber cropping systems, application of soil amendments significantly improved soil organic matter, total nitrogen content, and the α-diversity of rhizosphere microbial communities, resulting in a 20.39% increase in cucumber dry biomass^18^.

Despite the recognized benefits of organic fertilizers and the potential of soil conditioners, there remains a significant knowledge gap regarding the synergistic effects of their combined application, particularly in mitigating continuous cropping obstacles in protected cowpea production. Specifically, comprehensive understanding of how such combined treatments influence the soil microbiome and physicochemical properties in long-term monoculture systems is limited. To address this, this study hypothesized that the combined application of organic fertilizers and soil conditioners would synergistically improve soil health and cowpea productivity more effectively than individual treatments. We investigated their effects on soil fertility-related parameters and employed high-throughput sequencing to comprehensively characterize the resulting changes in soil microbial community structure and diversity in rhizosphere soils under long-term protected cowpea monoculture. This research aims to elucidate the influence of continuous cropping on microbial communities, evaluate the effectiveness of organic fertilizer and soil conditioning amendments, and provide technical strategies and theoretical foundations for mitigating continuous cropping obstacles and restoring soil function in protected agricultural systems.

## Materials and methods

### Site description

**Fig. 1:**
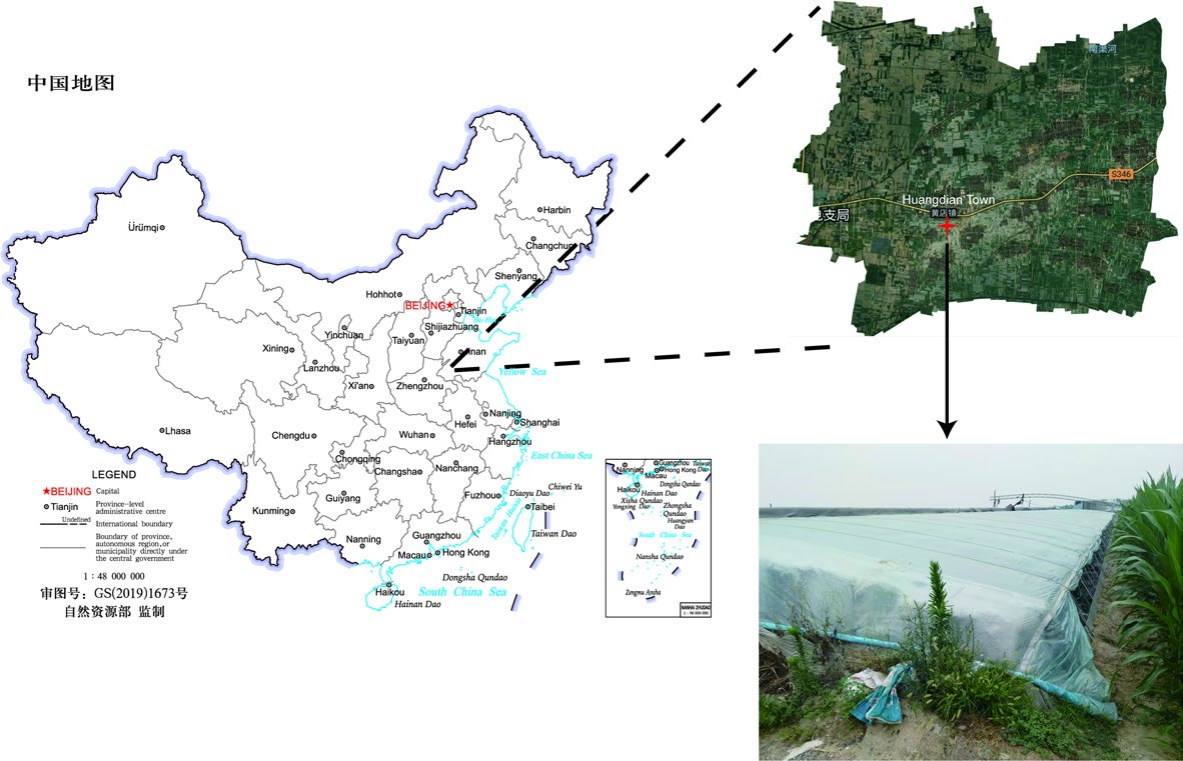
Site location of the experiment. This map is based on the standard map with approval number GS (2019)1673 downloaded from the website of the Standard Map Service of the Ministry of Natural Resources of China (http://bzdt.ch.mnr.gov.cn/index.html). The base map boundaries have not been modified.

The experiment was conducted in a sunlight greenhouse (35°07ʹ27ʹʹN, 115°26ʹ96ʹʹE) at a vegetable planting base in Huangdian Town, Dingtao District, Heze City, Shandong Province, China (Fig. 1). The experimental site was selected from a greenhouse that had been used continuously for vegetable cultivation for twenty-five years, with five consecutive years of facility-based cowpea cultivation. The soil type in the experimental area is loam, classified as fluvo-aquic soil, with a pH range between 5 and 8. This region has a warm temperate monsoon continental climate, with an average annual temperature of 13.9 °C and an average annual precipitation of 663.0 mm. The frost-free period lasts for 209 days.

### Experimental design

There were four treatments in the experiment: no fertilizer application (NF), chemical fertilizer application (F), chemical fertilizer + organic fertilizer (FO), and chemical fertilizer + organic fertilizer + soil conditioner (FOS). The chemical fertilizer was a potassium sulfate fertilizer with a total nutrient content ≥ 51%, formulated as N–phosphorus pentoxide (P₂O₅)–potassium oxide (K₂O) = 17–17–17 + trace elements. The organic fertilizer used in the experiment was fermented rabbit manure purchased from Shandong Dili Animal Husbandry Technology Co., Ltd. (Heze, China). This manure contained 40.7% OM, with nutrient contents of 1.58% N, 1.47% P₂O₅, and 0.41% K₂O. The soil conditioner applied in the trial contained ≥ 45% OM and ≥ 5.0 × 10⁸ colony-forming units/g of viable microorganisms. The primary microbial components included *Bacillus subtilis*, *B. licheniformis*, and *Trichoderma harzianum*. In addition, this manure contained ≥ 1 mg/g of polysaccharides and was supplemented with potassium humate derived from mineral sources. The chemical fertilizer and soil conditioner were purchased from Heze Jinzhengda Ecological Engineering Co., Ltd. (Heze, China). The experimental layout is provided in Table 1. All fertilizers and soil conditioners were applied as basal applications and thoroughly mixed into the top 20 cm of soil prior to planting.

The cowpea seeds used in the experiment were of the “Qinglong” cultivar, purchased from Shenyang Dapeng Vegetable Seed Sales Company (Shenyang, China), without any specific permission requirement. This variety is highly heat-tolerant, producing pods with a bright green color that remain unchanged in appearance and length even at temperatures exceeding 35 °C. Additionally, this variety is known for its stable yield performance. The experimental soil had a pH range of 7.5–8.5, classified as neutral soil. Each plot encompassed an area of 24 m² and was bordered by earthen ridges measuring 40 cm in width and 30 cm in height. The ridge spacing was set at 55 cm, and each ridge was 50 cm wide and 40 cm high. Within the ridges, individual plants were planted 25 cm apart, with row spacings of 50 cm. Each ridge contained two rows, and two ridges were planted per plot. Four to five seeds were sown per planting hole. Each treatment was replicated three times and arranged in a randomized block design. The soil in this trial was collected from greenhouses that had undergone five consecutive protected cowpea cropping cycles, and it was used to conduct a comparative study under continuous cropping conditions.

After comprehensively considering the effects of multiple factors such as biomass yield, quality, and soil indices, only the FO and FOS treatments were analyzed for microbial communities to specifically evaluate the effects of organic amendments and soil amendments on the soil microbiome. Soil samples were collected from the FO and FOS treatments at three key protected cowpea growth stages: seedling stage (10 days after planting), vegetative growth stage (40 days), and harvest stage (70 days). Soil samples were collected from the solar greenhouse using the five-point sampling method. Composite samples were collected from the 0 to 20 cm soil layer and used as experimental material. Each sampling point was collected in triplicate.

**Table 1:** The application amount of different kinds of fertilizer.

Fertilizer	Fertilization method	Fertilizer, kg/ha	Organic fertilizer, kg/ha	Soil conditioner, kg/ha
NF	No fertilization	0	0	0
F	Chemical fertilizer only	1350	0	0
FO	Chemical fertilizer + organic fertilizer	1350	9000	0
FOS	Chemical fertilizer + organic fertilizer + soil conditioner	1350	9000	1125

The fertilizer application rate is calculated based on the total amount per ha per year.

### Determination of nutritive values, soil physics, and mineral elements

The AOAC method was used to determine protein, fat, carbohydrate, and dietary fiber contents, as well as the energy of protected cowpea^19^. Soil pH and electrical conductivity (EC) were determined in agreement with SSSA methods^20^. The soil total nitrogen (TN), available nitrogen (AN), available phosphorus (AP), available potassium (AK), and OM were measured using a previously described method^21^. Slowly available potassium (SK) was measured using a soil nutrient analyzer (SL-TF16, Zhengzhou Silan Instrument Co., Ltd.). The soil mineral element contents, including calcium (Ca), magnesium (Mg), iron (Fe), manganese (Mn), copper (Cu), zinc (Zn), and boron (B) were determined using atomic absorption spectroscopy after digestion according to the method described by Li et al.^22^.

### Determination of soil enzyme activities

The activities of soil urease (UE), sucrase (SC), dehydrogenase (DHO), and catalase (CAT) were measured using a previously described method^21^. Soil acid phosphatase (ACP) activity was determined using the disodium phenyl phosphate colorimetric method^23^. Soil polyphenol oxidase (PPO) was detected using the autoxidation of pyrogallol method, and soil protease (PT) was determined via casein hydrolysis^24^. Soil nitrate reductase (NR) activity was measured using the phenanthroline–sulfuric acid colorimetric method^25^.

### DNA extraction

The CTAB method was employed to extract DNA from the soil samples. A 1% agarose gel was prepared, and the quality of extracted DNA was assessed using electrophoresis. DNA concentration and purity were measured using a micro-volume spectrophotometer. Then, the samples were diluted to a concentration of 1 ng/µL and stored at − 80 °C for future use.

### Soil bacterial polymerase chain reaction (PCR) amplification and 16S rDNA gene sequencing

The primers 338 F and 806R were used to amplify the V4-V5 hypervariable region using PCR. The PCR products were purified and subjected to high-throughput sequencing using an Illumina MiSeq PE300 platform by Shenzhen Weishengtai Technology Co., Ltd. The template for sequencing was diluted genomic DNA.

### Soil fungal PCR amplification and ITS gene sequencing

Using diluted genomic DNA as the template and the primers ITS1F and ITS2R, PCR amplification was conducted, and the products were sent to Shenzhen Weishengtai Technology Co., Ltd. for high-throughput sequencing using an Illumina MiSeq PE300 platform.

### Data analysis

Alpha diversity indices, including Chao1, Shannon, Simpson, and ACE, were calculated using QIIME v1.9.1. Redundancy analysis (RDA) and principal factor evaluations were performed using Canoco v5.0 to assess sample reproducibility and identify the key influencing factors. SPSS v28.0 was used to conduct correlation analyses, and independent sample t-tests were conducted to evaluate the significance of differences between treatments. The Mantel test, performed in R (v3.6.2), was employed to elucidate the correlations among soil physicochemical properties, cowpea quality traits, and soil bacterial and fungal communities. Key visualizations were generated using Origin 2018 to illustrate critical aspects of these analyses.

### Ethical statement

Statement on guidelines as experimental research and field studies on cowpea. Experimental research and field studies on cowpea (either cultivated and wide), including the collection of cowpeas comply with relevant institutional, national, and international guidelines and legislation, and these studies comply with local and national regulations. The measurement process of microorganism and rare earth content will not affect the local soil microorganism and ecological environment, etc. During the process of experiment, aseptic sampling was carried out to avoid contamination, and the research was evaluated and agreed by the environmental protection authorities of the local government.

## Results

### Effects of organic fertilizer and soil conditioner on the yield of protected cowpea

After harvest, the yield-related traits of cowpea, including single pod length, single pod weight, and yield per plant, were evaluated. All fertilizer treatments resulted in a significant increase compared with NF (*p* < 0.05). Among the treatments, the FOS treatment produced the highest yield. The average yield of the NF group was 16,058.25 kg/ha, while the yield of the FOS treatment increased to 76,953.90 kg/ha, indicating a substantial improvement (Table 2).

**Table 2:** Yield of protected Cowpea under different fertilizer treatments.

Treatment	Single pod length, cm	Single pod weight, g	Single plant yield, kg	Yield per ha, kg
NF	61.04 ± 1.21 D	19.33 ± 0.46 D	0.13 ± 0.10 D	16058.25 ± 1297.99 D
F	73.28 ± 2.10 C	22.58 ± 1.09 C	0.21 ± 0.10 C	30527.70 ± 883.61 C
FO	80.05 ± 2.56 B	25.54 ± 2.35 B	0.49 ± 0.15 B	44104.95 ± 1837.65 B
FOS	89.33 ± 3.18 A	28.31 ± 1.23 A	0.57 ± 0.19 A	76953.90 ± 1970.55 A

Statistical significance was set at a level of *p* < 0.05 using Duncan’s multiple range tests. The same letter in table represents no significant difference.

### Effects of organic fertilizer and soil conditioner on the quality of protected cowpea

Given the significantly higher yields observed in the FO and FOS treatment groups (Table 2), and to efficiently characterize the most promising quality improvements, subsequent detailed quality analyses were focused on these two treatments. To further evaluate the effects of combining organic fertilizer with a soil conditioner on cowpea quality, several nutritional parameters were measured after pod harvest, including protein, fat, dietary fiber, and carbohydrate contents, as well as energy value (Table 3). The results showed that the FOS group exhibited extremely significant increases in protein and carbohydrate contents and energy value, while the fat content decreased significantly compared with the FO group (*p* < 0.05). These findings indicate that applying a soil conditioner can further improve the nutritional quality of protected cowpea.

**Table 3:** The effect of applying organic fertilizer and soil conditioner on the quality of protected cowpea.

Treatment	Protein content, g/100 g	Fat content, g/100 g	Dietary fiber, g/100 g	Carbohydrate, g/100 g	Energy, KJ/100 g
FO	2.26 ± 0.06 B	0.11 ± 0.02 A	3.41 ± 0.22 A	4.62 ± 0.13 B	158.35 ± 6.07 B
FOS	3.00 ± 0.07 A	0.02 ± 0.03 B	3.68 ± 0.19 A	7.98 ± 0.21 A	198.53 ± 5.45 A

Statistical significance was set at a level of *p* < 0.05 using Duncan’s multiple range tests. The same letter in table represents no significant difference.

### Effects of organic fertilizer and soil conditioner on soil pH and nutrient content in continuous cropping of protected cowpea

To further investigate the effects of combining organic fertilizer with a soil conditioner on soils subjected to continuous cowpea cultivation, soil pH and nutrient contents were analyzed at different growth stages following five consecutive crop cycles in the FO and FOS treatment groups (Table 4). The soil pH in the FOS group decreased significantly by 5.4% compared with the FO groups, reaching approximately 7.5. This pH range is conducive to the growth of the majority of microorganisms involved in OM decomposition under neutral conditions, thus enhancing microbial activity and promoting OM mineralization. As shown in Table 4, all FOS groups exhibited significantly higher soil OM contents compared with the FO groups (*p* < 0.05). Additionally, the FOS groups demonstrated substantially increased levels of AP and AK, particularly at 40 days after sowing, when AP reached 27.53 mg/kg, and AK peaked at 563.92 mg/kg. In contrast, soil EC varied across treatments, with the FOS treatment at 40 days displaying a moderate EC value, indicating balanced salt accumulation.

**Table 4:** Soil chemical properties and pH of different treatments.

Treatment	pH	Total nitrogen (TN), g/kg	Available nitrogen (AN), mg/kg	Available phosphorus (AP), mg/kg	Organic matter (OM), g/kg	Available potassium (AK), mg/kg	Slowly available potassium (SK), mg/kg	Electrical conductivity (EC), µS/cm
FO-10 d	7.86 ± 0.03 A	0.78 ± 0.04 C	51.24 ± 2.12 C	18.99 ± 1.24 B	9.08 ± 0.12 C	512.23 ± 8.59 C	617.31 ± 3.15 D	551.00 ± 3.00 C
FO-40 d	7.89 ± 0.05 A	0.84 ± 0.04 BC	43.34 ± 2.34 C	18.15 ± 1.43 B	10.28 ± 0.22 C	493.98 ± 9.44 D	632.68 ± 2.36 C	848.00 ± 5.00 A
FO-70 d	7.99 ± 0.04 A	0.89 ± 0.03 AB	44.51 ± 3.84 C	20.07 ± 1.5 B	10.65 ± 0.30 C	411.78 ± 3.37 E	605.92 ± 3.02 E	624.00 ± 6.00 B
FOS-10 d	7.45 ± 0.02 B	0.95 ± 0.04 A	76.44 ± 4.60 B	21.13 ± 2.17 B	13.76 ± 0.15 B	537.68 ± 6.16 B	614.28 ± 2.88 DE	503.00 ± 7.00 D
FOS-40 d	7.46 ± 0.06 B	0.94 ± 0.04 A	80.65 ± 4.78 B	27.53 ± 1.67 A	17.78 ± 0.19 A	563.92 ± 3.23 A	729.85 ± 4.25 A	618.00 ± 4.00 B
FOS-70 d	7.55 ± 0.07 B	0.97 ± 0.03 A	90.79 ± 2.24 A	27.37 ± 4.18 A	15.08 ± 0.17 AB	548.57 ± 2.45 AB	658.17 ± 3.92 B	475.00 ± 6.00 E

FO-10 d, FO-40 d and FO-70 d are soil samples from the FO groups taken during the seedling stage, vegetative growth stage, and harvest stage, respectively; FOS-10 d, FOS-40 d and FOS-70 d are soil samples from the FOS groups taken during the seedling stage, vegetative growth stage, and harvest stage, respectively. Statistical significance was set at a level of *p* < 0.05 using Duncan’s multiple range tests. The same letter in table represents no significant difference.

The mineral element content in the soil was subsequently analyzed under different treatments (Table 5). The FOS treatment did not significantly affect the levels of Ca, Fe, Mn, Cu, Zn, or B in the soil compared with the FO treatment (*p* > 0.05). However, significant increases were observed in the concentrations of Mg, especially in the FOS 10-day group, which contained the highest Mg content (*p* < 0.05). The enrichment of these mineral nutrients may contribute to improved soil fertility and partially influence protected cowpea quality.

**Table 5:** Soil mineral element content under different treatments.

Treatment	Ca, g/kg	Mg, g/kg	Fe, g/kg	Mn, mg/kg	Cu, mg/kg	Zn, mg/kg	B, mg/kg
FO-10 d	25.13 ± 0.76 B	2.95 ± 0.02 B	21.89 ± 1.16 A	464.46 ± 9.26 A	15.07 ± 0.56 B	59.20 ± 5.31 A	51.40 ± 2.03 A
FO-40 d	27.90 ± 0.32 A	2.85 ± 0.0.06 BC	22.08 ± 2.31 A	465.67 ± 8.12 A	16.21 ± 0.62 AB	57.69 ± 4.29 A	51.79 ± 1.89 A
FO-70 d	28.53 ± 0.89 A	2.11 ± 0.05 E	21.90 ± 1.56 A	468.22 ± 7.66 A	16.84 ± 0.32 A	56.49 ± 3.15 A	50.04 ± 2.14 A
FOS-10 d	28.41 ± 0.25 A	3.34 ± 0.09 A	23.93 ± 3.08 A	473.90 ± 8.08 A	16.58 ± 0.46 A	66.15 ± 2.68 A	53.82 ± 3.31 A
FOS-40 d	28.21 ± 0.68 A	2.78 ± 0.06 C	21.61 ± 2.54 A	467.30 ± 7.52 A	16.73 ± 0.55 A	63.25 ± 4.20 A	50.52 ± 2.84 A
FOS-70 d	27.87 ± 0.41 A	2.53 ± 0.07 D	21.70 ± 1.86 A	455.48 ± 9.4 A	15.96 ± 0.68 AB	58.05 ± 3.95 A	50.90 ± 1.75 A

Statistical significance was set at a level of *p* < 0.05 using Duncan’s multiple range tests. The same letter in table represents no significant difference.

### Effects of organic fertilizer and soil conditioner on soil enzyme activities in continuous cropping substrate of protected cowpea

As shown in Table 6, among the eight enzymes examined, the activities of four enzymes (excluding DHO, PPO, PT, and NR) exhibited differential changes across various developmental stages of cowpea under both FO and FOS treatments. When comparing enzyme activities between the FO and FOS treatment groups at the same growth stage, it was observed that, with the exception of NR, which showed no significant change, the activities of the remaining seven enzymes were significantly higher in the FOS-treated group than in the FO-treated group. Specifically, in the FOS treatment at 70 days, the activities of UE, SC, and ACP increased by 55.81%, 35.99%, and 109.00%, respectively, compared to those in the FO treatment group.

**Table 6:** Effects of different fertilization treatments on soil enzyme activities.

Treatment	Urease (UE), mg/d/g	Sucrase (SC), mg/d/g	Acid phosphatase (ACP), nmol/h/g	Dehydrogenase (DHO), µg/d/g	Catalase (CAT), µmol/d/g	Polyphenol oxidase (PPO), nmol/h/g	Protease (PT), mg/d/g	Nitrate reductase (NR), µmol/d/g
FO-10 d	6.23 ± 0.07 D	3.97 ± 0.18 C	240.49 ± 9.58 C	40.94 ± 1.87 B	27.22 ± 0.25 AB	553.34 ± 9.73 AB	2.61 ± 0.06 C	0.17 ± 0.02 B
FO-40 d	6.09 ± 0.08 D	3.62 ± 0.19 CD	195.73 ± 8.24 D	41.89 ± 2.23 B	24.10 ± 0.29 D	512.93 ± 10.94 B	2.56 ± 0.15 C	0.22 ± 0.04 AB
FO-70 d	5.16 ± 0.12 E	3.39 ± 0.09 D	172.31 ± 6.55 E	39.05 ± 1.36 B	22.30 ± 0.34 E	508.79 ± 18.28 B	2.61 ± 0.10 C	0.19 ± 0.01 AB
FOS-10 d	7.06 ± 0.09 C	4.00 ± 0.12 C	249.01 ± 6.10 C	48.53 ± 0.98 A	27.88 ± 0.38 A	585.81 ± 17.96 A	3.57 ± 0.09 B	0.20 ± 0.03 AB
FOS-40 d	7.67 ± 0.11 B	5.52 ± 0.15 A	307.85 ± 7.21 B	50.42 ± 2.41 A	26.27 ± 0.44 C	589.15 ± 19.78 A	4.00 ± 0.17 A	0.25 ± 0.02 A
FOS-70 d	8.04 ± 0.07 A	4.61 ± 0.16 B	360.13 ± 8.87 A	47.58 ± 1.05 A	26.93 ± 0.21 BC	594.55 ± 22.49 A	4.11 ± 0.08 A	0.25 ± 0.03 A

Statistical significance was set at a level of *p* < 0.05 using Duncan’s multiple range tests. The same letter in table represents no significant difference.

### Effects of organic fertilizer combined with soil conditioner on soil microbial community structure in continuous cropping protected cowpea

**Fig. 2:**
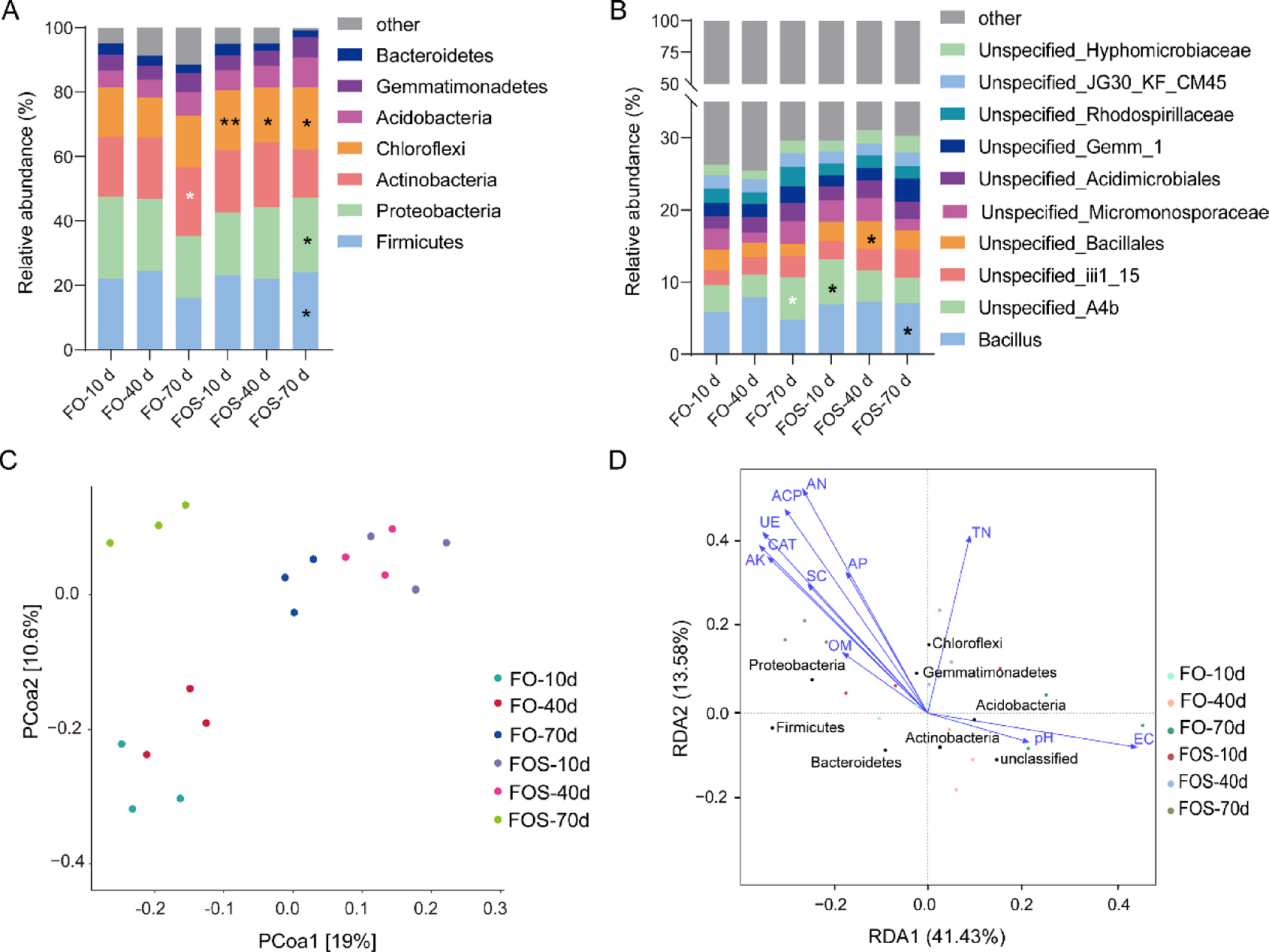
Bacterial community composition, principal coordinate analysis (PCoA) and redundancy analysis (RDA) of soil samples. (**A**) Relative abundance of bacterial phyla. (**B**) Relative abundance of dominant bacterial genera. (**C**) PCoA of bacterial communities based on 18 soil samples. (**D**) RDA illustrates the relationships between soil bacterial phyla, soil physicochemical properties and enzyme activities.

The dominant bacterial phyla in the soil of protected cowpea included Firmicutes, Proteobacteria, Actinobacteria, Chloroflexi, Acidobacteria, Gemmatimonadetes, and Bacteroidetes, collectively accounting for greater than 95% of the total relative abundance (Fig. 2A). The FOS groups significantly increased the relative abundance of Chloroflexi by 20.96% compared with the FO groups at the seedling and vegetative growth stages. At the harvest stage, notable increases in Firmicutes (49.43%), Proteobacteria (19.05%), and Chloroflexi (20.45%) were observed in the FOS group (Fig. 2A). At the bacterial genus level, *Bacillus* exhibited a significant increase in relative abundance in the FOS groups at the harvest stage, with a 45.83% increase compared with the FO groups (Fig. 2B).

The assessment of soil microbial community structure was conducted using alpha diversity indices, including Chao1, Shannon, and Simpson metrics. Bacterial and fungal alpha diversity were measured in soil samples collected at 10, 40, and 70 days from the FO and FOS treatment groups (Supplementary Table 1). The results showed that, for bacteria, the FOS groups exhibited slightly higher Chao1 and Shannon indices compared with the FO groups at each time point, particularly at 70 days, where the FOS group showed the highest Shannon index (9.64 ± 0.06) and Chao1 richness (1350.92 ± 27.47) values. The Simpson index remained consistently high across all treatments, indicating a stable bacterial community evenness.

PCoA analyses demonstrated beta diversity, where samples from the same treatment clustered and samples from all six treatments were distinguishable. The first and second principal coordinates in combination explained 29.6% of the variation in community structure (Fig. 2C). RDA was employed to further elucidate the relationship between bacterial phyla and soil physicochemical properties (*p* < 0.05) (Fig. 2D). The first two axes in the RDA in combination explained 55.01% of the total variation in soil bacterial community structure. The dominant bacterial phyla were Proteobacteria, Chloroflexi, and Gemmatimonadetes, exhibiting significant positive correlations with OM, TN, AN, AK, AP, SC, UE, ACP, and CAT and negative correlations with pH and EC. Conversely, Actinobacteria was positively correlated with pH and EC but negatively correlated with all other measured indicators. This pattern of correlation suggests that Actinobacteria’s abundance may be associated with specific soil conditions that influence nutrient cycling, rather than directly indicating an unfavorable impact on cowpea growth (Fig. 2D).

**Fig. 3:**
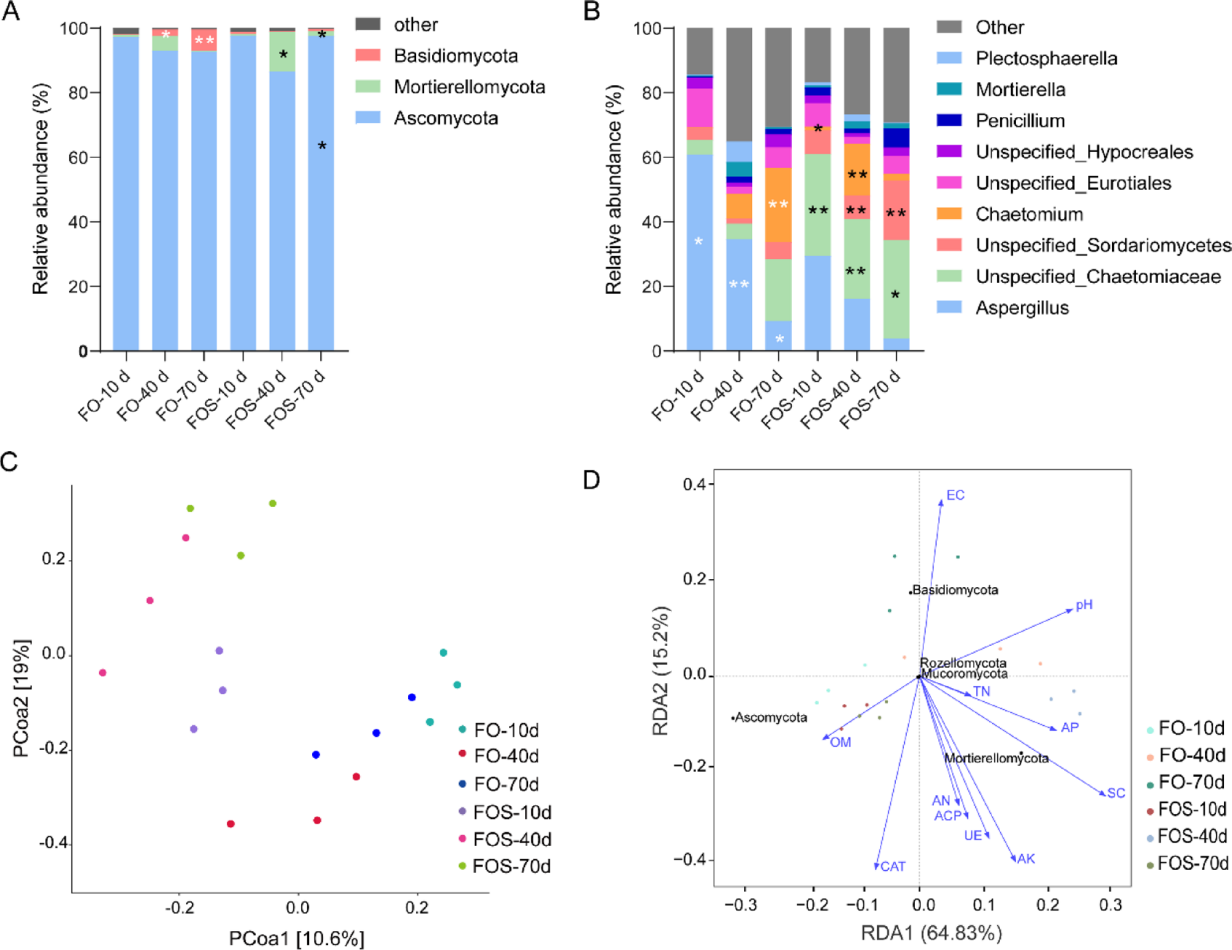
Fungal community composition, PCoA and RDA of soil samples. (**A**) Relative abundance of fungal phyla. (**B**) Relative abundance of dominant fungal genera. (**C**) PCoA of fungal communities based on 18 soil samples. (**D**) RDA illustrates the relationships between soil fungal phyla, soil physicochemical properties and enzyme activities.

For the fungal community, the dominant phyla were Ascomycota, Mortierellomycota, and Basidiomycota, in combination representing greater than 98% of the fungal population. During the vegetative growth stage, Basidiomycota exhibited a marked decrease in relative abundance (− 85.22%). In contrast, the abundance of Mortierellomycota increased significantly by 160.86% (Fig. 3A). At the harvest stage, fungal community shifts were most pronounced: Ascomycota increased by 4.92%, while Basidiomycota decreased dramatically by 89.01%. Notably, Mortierellomycota surged by 552.17%, representing a substantial proportional increase from its original relative abundance in response to the FOS treatment (Fig. 3A). For fungal genera, the relative abundance of *Aspergillus* consistently decreased across all three stages by 51.54%, 53.13%, and 58.23%, respectively. In contrast, the genus *Chaetomium* (represented as *unspecified_Chaetomiac*) displayed significant increases of 573.91%, 414.16%, and 58.85% at the seedling, vegetative, and harvest stages, respectively (Fig. 3B). Furthermore, the FOS treatment consistently enhanced fungal alpha diversity across all sampling points. Specifically, the 70-day FOS group exhibited the highest fungal Shannon diversity index (5.00 ± 0.30) along with relatively high Chao1 richness (125.00 ± 10.02) compared with the FO treatment groups (Supplementary Table 1).

Similarly, the PCoA analyses revealed that the first and second principal coordinates accounted for 29.6% of the total variation in fungal community structure (Fig. 3C). The first two axes of the RDA in combination explained 80.03% of the total variation in soil bacterial community structure. (Fig. 3D). The dominant fungal phylum Ascomycota demonstrated positive correlations with OM, AN, AK, CAT, UE, and ACP and negative correlations with pH, EC, TN, AP, and SC. Similarly, Mortierellomycota exhibited negative associations with OM and EC but positive relationships with other physicochemical parameters. Notably, Basidiomycota was positively correlated with EC and pH and negatively correlated with all other measured indicators. This correlative pattern suggests that Basidiomycota may be associated with soil conditions that are less favorable for overall soil health or cowpea performance, rather than directly causing adverse effects (Fig. 3D).

### Correlations between soil microbial community structure, soil fertility factors, and the quality of protected cowpea

**Fig. 4:**
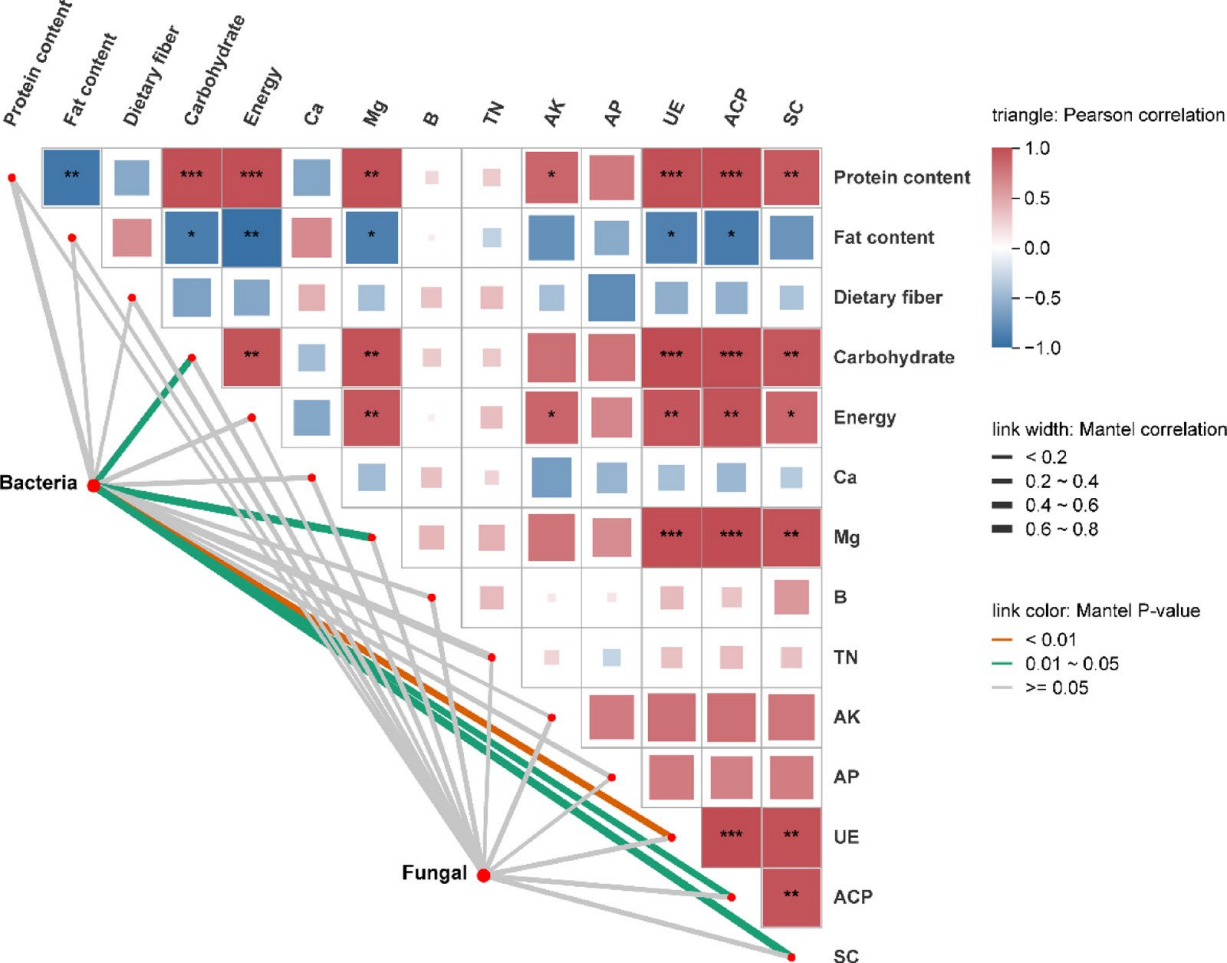
The relationship between physicochemical factors and the quality of cowpeas with bacteria and fungi was obtained by the Mantel test.

Mantel tests were conducted to elucidate the correlations among soil physicochemical properties, cowpea quality parameters, and the abundances of bacterial and fungal communities at the harvest stage. The results showed that bacterial operational taxonomic unit (OTU) abundance was significantly positively correlated with ACP, CAT, pH, OM, Mg, and carbohydrate content (Fig. 4). Additionally, the OTU abundance of fungi did not indicate a significant positive correlation with the quality indicators of cowpeas, as well as the physicochemical properties of the soil and enzyme activities. Meanwhile, UE, ACP, SC, and Mg display a significant positive correlation with cowpea protein and carbohydrate contents, as well as energy (*p* < 0.05). In contrast, the fat content displays a significant negative correlation with UE, ACP, and Mg (*p* < 0.05). These findings indicate that applying organic fertilizers may enhance the activity levels of enzymes and promote the decomposition of inorganic nutrients in the soil, thus improving soil fertility and ultimately enhancing cowpea quality.

## Discussion

### Effects of combined organic fertilizer and soil conditioner on soil fertility factors in continuous cropping systems for protected cowpea

Soil nutrients and organic matter are crucial for healthy plant growth. Soil degradation caused by continuous cropping obstacles severely restricts the development of facility-based agriculture, and this issue is becoming increasingly prominent in the cowpea farming industry. In recent years, studies have indicated that the combined application of organic fertilizer and soil conditioners can effectively mitigate soil degradation and has been widely adopted in the cultivation of vegetables such as tomatoes^26^, cucumbers^18^ and garlic^27^. This study found that the combined application of organic fertilizer and soil conditioners effectively lowers the pH of continuous cropping soil for cowpeas, which aligns with the findings of Chen et al. (2025), who reported that microbial organic fertilizer reduces the pH of soil used for continuous cropping of *Pinellia ternata*^28^. The decrease in soil pH may be attributed to the activity of microbial communities in the soil conditioner, which produce pyruvic acid that is subsequently converted into organic acids such as lactic acid and acetic acid, thereby reducing soil acidity^29^. Soil enzyme activity serves as a key catalyst for nutrient cycling and is a sensitive indicator of soil health^30^. Our results demonstrate that soil conditioners can induce increased activity in most enzymes, including UE, SC, and ACP. It is hypothesized that soil conditioners may create a more suitable microenvironment for enzyme stability and function by improving soil aggregate structure and regulating pH levels^16^. Specifically, sucrase activity is closely related to carbon source metabolism, while urease and phosphatase are involved in nitrogen cycling and the mineralization of organic phosphorus, respectively^31–33^. The enhanced activity of these enzymes effectively promotes the decomposition of organic fertilizers and the accumulation of available nitrogen and phosphorus in the soil. Consistent with this, soil conditioner treatment significantly increased the content of OM, AN, AP and AK. Therefore, our findings indicate that the combined application of organic fertilizer and soil conditioners effectively improves the overall quality of continuous cropping soil for cowpeas, thereby enhancing both yield and quality.

### Effects of combined application of organic fertilizer and soil conditioner on the soil microbial community structure in protected cowpea under continuous cropping

Soil microorganisms play a crucial role in maintaining soil vitality and the soil ecosystem. The soil microbial environment can directly affect the abundance, activity, diversity, and community structure of these microorganisms^34,35^. After applying soil conditioner, Chloroflexi, Proteobacteria, Mortierellomycota, and Bacillus exhibited highly significant positive correlations with fertilizer-related factors. These taxa were entirely distinct from the dominant microbial communities observed in the control group, indicating that adding soil conditioner substantially altered the soil microbial community structure. The increases in Chloroflexi and Proteobacteria suggest that the FOS treatment created a nutrient-rich environment conducive to the rapid breakdown of organic amendments. The notable increase in Bacillus is particularly significant, as many Bacillus species are recognized as plant growth-promoting rhizobacteria capable of nutrient solubilization, nitrogen fixation, and even biocontrol against plant pathogens^36^. For the fungal community, the dramatic surge in Mortierellomycota (up to 552.17% increase) under FOS treatment, alongside a significant decrease in Basidiomycota, is a key finding. Mortierellomycota are saprophytic fungi known for their rapid decomposition of organic matter and ability to produce plant growth-promoting substances^37^. Their pronounced increase strongly indicates that the FOS treatment, with its rich organic inputs and potentially specific growth factors, created a highly favorable niche for these fast-acting decomposers, contributing to accelerated turnover of organic matter and nutrient release. Conversely, Actinobacteria showed a negative correlation with improved soil fertility indicators (OM, available nutrients) and enzyme activities, and Basidiomycota was positively correlated with EC and pH and negatively correlated with other measured indicators. These correlative patterns suggest that these phyla may be associated with soil conditions that are less favorable for overall soil health or cowpea performance, or they may be outcompeted in the nutrient-rich, biologically active environment fostered by the FOS treatment, rather than directly indicating an unfavorable impact on cowpea growth. Notably, soil improvement is a long-term process and dramatic enhancements in soil properties cannot be expected in the short term.

### Effects of combined application of organic fertilizer and soil conditioner on the yield and quality of protected cowpea

Previous studies have confirmed that using organic fertilizers and microbial inoculants has a significantly positive impact on plant growth. For example, these products can effectively promote the development of a plethora of crops, including tomato, garlic, cucumber, lettuce, yam, and wax gourd^38–43^. However, certain studies have indicated that microbial inoculants alone may not significantly accelerate plant growth^44^. In this study, the soil conditioner applied contained biocontrol agents such as *B. subtilis*, *B. licheniformis*, and *T. harzianum*. When used in combination with organic fertilizer, this treatment markedly improved the yield and quality of protected cowpea, resulting in a high-yield, high-quality vegetable characterized by high protein and low-fat contents. These findings further demonstrate that applying soil conditioner enhances the microbial ecosystem in the rhizosphere of cowpea, promotes the proliferation of beneficial bacteria, increases soil enzyme activity, and reduces nutrient losses, ultimately contributing to the robust growth and development of the crop. The synergistic interaction between organic amendments, specific microbial inoculants, and the resulting shifts in the native soil microbiome created an optimal environment for protected cowpea growth and nutrient uptake.

In summary, this study reveals the role and potential mechanisms of soil conditioner combined with organic fertilizer in alleviating the continuous cropping obstacles of cowpea, which holds significant importance for the development of the cowpea industry. However, the absence of NF and F treatment groups in the analysis of soil and microbial indicators, along with the lack of multi-year replicated experiments and measurement of cowpea rhizosphere secretions, has limited a more comprehensive understanding of the mechanisms by which soil conditioner enhances the yield and quality of continuously cropped cowpea. These limitations also suggest avenues for future research.

## Conclusion

**Fig. 5:**
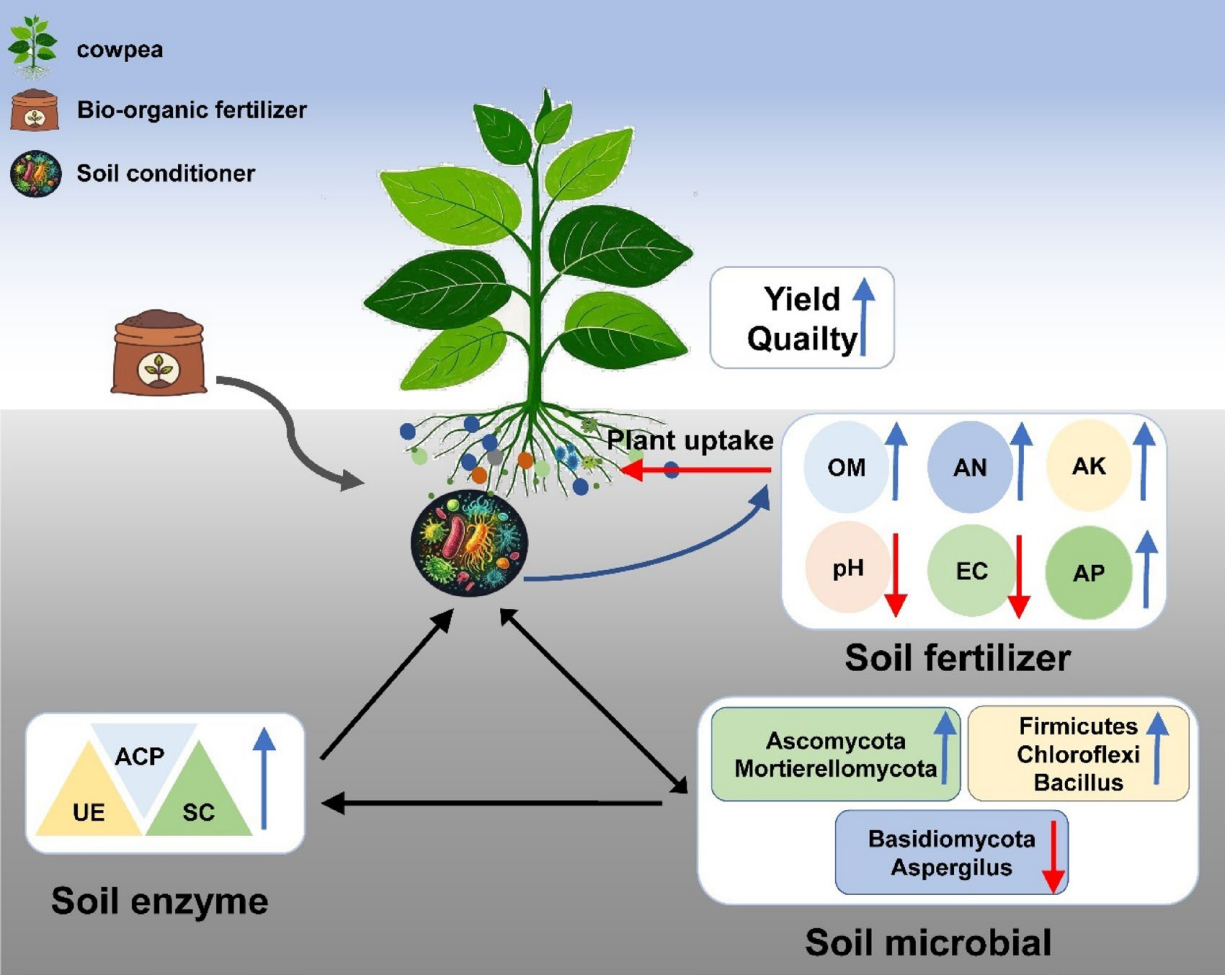
Mechanism model of high yield and high-quality cultivation techniques for protected cowpea resistant to repetitive crops.

The decline in yield and quality of cowpea caused by continuous cropping obstacles has become a major constraint in cowpea cultivation. This study investigated the combined application of organic fertilizer and soil conditioner to alleviate continuous cropping obstacles in cowpea. The results demonstrated that the addition of soil conditioner contributed to reducing soil pH, increasing the activities of key soil enzymes such as UE, ACP, and SC, and promoting the content of OM, AN, AK, and AP. Furthermore, it effectively enhanced the diversity of soil bacteria and fungi, as well as the abundance of beneficial microorganisms. Thus, the combined application of organic fertilizer and soil conditioner can effectively improve the quality of continuously cropped soil, thereby enhancing the yield and quality of cowpea (Fig. 5). This study provides a reference for the scientific cultivation of cowpea.

## Supplementary Information

Below is the link to the electronic supplementary material.

Supplementary Material 1

## Data Availability

Sequence data that support the findings of this study have been deposited in the NCBI SRA with the primary accession code PRJNA1297432.
